# Silencing *Folylpolyglutamate Synthetase1* (*FPGS1*) in Switchgrass (*Panicum virgatum* L.) Improves Lignocellulosic Biofuel Production

**DOI:** 10.3389/fpls.2020.00843

**Published:** 2020-06-19

**Authors:** Mitra Mazarei, Holly L. Baxter, Avinash Srivastava, Guifen Li, Hongli Xie, Alexandru Dumitrache, Miguel Rodriguez, Jace M. Natzke, Ji-Yi Zhang, Geoffrey B. Turner, Robert W. Sykes, Mark F. Davis, Michael K. Udvardi, Zeng-Yu Wang, Brian H. Davison, Elison B. Blancaflor, Yuhong Tang, Charles Neal Stewart

**Affiliations:** ^1^Department of Plant Sciences, The University of Tennessee, Knoxville, TN, United States; ^2^BioEnergy Science Center, Oak Ridge National Laboratory, Oak Ridge, TN, United States; ^3^The Center for Bioenergy Innovation, Oak Ridge National Laboratory, Oak Ridge, TN, United States; ^4^Noble Research Institute, Ardmore, OK, United States; ^5^Biosciences Division, Oak Ridge National Laboratory, Oak Ridge, TN, United States; ^6^National Renewable Energy Laboratory, Golden, CO, United States

**Keywords:** folylpolyglutamate synthetase, switchgrass, RNAi-gene silencing, *PvFPGS1*, lignocellulosic, biofuel

## Abstract

Switchgrass (*Panicum virgatum* L.) is a lignocellulosic perennial grass with great potential in bioenergy field. Lignocellulosic bioenergy crops are mostly resistant to cell wall deconstruction, and therefore yield suboptimal levels of biofuel. The one-carbon pathway (also known as C1 metabolism) is critical for polymer methylation, including that of lignin and hemicelluloses in cell walls. Folylpolyglutamate synthetase (FPGS) catalyzes a biochemical reaction that leads to the formation of folylpolyglutamate, an important cofactor for many enzymes in the C1 pathway. In this study, the putatively novel switchgrass *PvFPGS1* gene was identified and its functional role in cell wall composition and biofuel production was examined by RNAi knockdown analysis. The *PvFPGS1*-downregulated plants were analyzed in the field over three growing seasons. Transgenic plants with the highest reduction in *PvFPGS1* expression grew slower and produced lower end-of-season biomass. Transgenic plants with low-to-moderate reduction in *PvFPGS1* transcript levels produced equivalent biomass as controls. There were no significant differences observed for lignin content and syringyl/guaiacyl lignin monomer ratio in the low-to-moderately reduced *PvFPGS1* transgenic lines compared with the controls. Similarly, sugar release efficiency was also not significantly different in these transgenic lines compared with the control lines. However, transgenic plants produced up to 18% more ethanol while maintaining congruent growth and biomass as non-transgenic controls. Severity of rust disease among transgenic and control lines were not different during the time course of the field experiments. Altogether, the unchanged lignin content and composition in the low-to-moderate *PvFPGS1*-downregulated lines may suggest that partial downregulation of *PvFPGS1* expression did not impact lignin biosynthesis in switchgrass. In conclusion, the manipulation of *PvFPGS1* expression in bioenergy crops may be useful to increase biofuel potential with no growth penalty or increased susceptibility to rust in feedstock.

## Introduction

The addition or removal of one-carbon units (C1 metabolism) is required for the synthesis and regulation of many biological compounds and metabolic processes. The synthesis of basic compounds and a range of methylated molecules in all organisms requires C1 metabolism (Appling, 1991). In plants, a balanced supply of C1 units is required for the synthesis of numerous plant secondary metabolites (e.g., lignin and phytohormones) and hemicellulose (Hanson and Roje, 2001; Eckardt, 2007; Urbanowicz et al., 2012). As the major sink of methyl units, lignin biosynthesis is affected by changes of enzymes in the C1 pathway (Shen et al., 2002; Tang et al., 2014; Li et al., 2015). Folylpolyglutamate synthetase (FPGS) catalyzes a biochemical reaction that leads to the formation of folylpolyglutamate, an important cofactor for many enzymes in the C1 pathway (Shane, 1989). In plant cells, folates, mainly as polyglutamylated byproducts, are found in chloroplasts, mitochondria, and cytosol (Rébeillé et al., 2006; Hanson and Gregory, 2011). In Arabidopsis, there are three genes each encoding FPGS isoforms that are localized to chloroplasts (FPGS1), mitochondria (FPGS2), and cytosol (FPGS3) (Ravanel et al., 2001).

Switchgrass (*Panicum virgatum* L.) is a lignocellulosic perennial grass known for its high yield of biomass, wide adaptability, and ability to grow on marginal soil conditions. These characteristics have made switchgrass a promising bioenergy feedstock (van der Weijde et al., 2013). One of the major problems with lignocellulosic crops is the resistance of the cell wall to deconstruction for efficient conversion into biofuels (known as biomass recalcitrance) (Himmel and Bayer, 2009). Cell wall lignin is one of the main causes of recalcitrance, which limits efficient conversion of biomass into biofuels (Chen and Dixon, 2007; David and Ragauskas, 2010). Cell wall components have been key targets to reduce feedstock recalcitrance; manipulating cell wall biosynthesis gene expression has been the primary strategy (Nelson et al., 2017; Biswal et al., 2018; Brandon and Scheller, 2020). Several studies with transgenic plants have been conducted in greenhouses under tightly controlled environmental conditions and the actively growing green tissue has been analyzed most often (Fu et al., 2011a, b, 2012; Xu et al., 2011; Shen et al., 2012, 2013; Liu et al., 2018). In contrast, end-of-season senesced tissue is most often used for biofuel production. Thus, greenhouse experiments may not be predictive of transgenic plants performance and recalcitrance of field-grown plants. Field-grown plants are exposed to a wider range of biotic and abiotic stresses not present in the greenhouse. Therefore, field experiments are especially important for modified plants to better predict agronomic performance across multiple growing seasons.

We previously showed that reduced lignin content and improved cell wall digestibility was observed in an Arabidopsis mutant with a disrupted *FPGS1* gene (Srivastava et al., 2015). We also showed that disruption in both FPGS1 and caffeoyl-CoA-3-*O*-methyltransferase (CCoAOMT), a lignin biosynthetic enzyme, resulted in further reduction in lignin content and improvement in cell wall digestibility in Arabidopsis (Xie et al., 2019). These studies prompted us to examine the possible role of FPGS in improving biofuel production for switchgrass.

In the present study, a novel switchgrass *PvFPGS1* gene was identified and its functional role was examined by downregulation using RNAi technology in switchgrass. A field experiment using multiple transgenic switchgrass lines downregulating *PvFPGS1* was conducted for three field growing seasons (2014–2016) to evaluate (i) *PvFPGS1* transcript levels, (ii) growth traits and biomass production, (iii) cell wall composition, sugar release, and conversion into biofuel, and (iv) susceptibility to rust disease.

## Materials and Methods

### Gene Identification

Using the *FPGS* cDNA sequence of Arabidopsis *AtFPGS1* (At5g05980), TBLASTN was used to identify the homologous gene sequences from switchgrass EST databases (Zhang et al., 2013) as well as from the draft switchgrass genome (*Panicum virgatum* v1.1 DOE-JGI) at Phytozome. A FPGS family gene tree was originally constructed using neighbor-joining in the software MEGA6 (Tamura et al., 2013) and a potential isolog of *AtFPGS1* was identified from switchgrass. A phylogeny tree for FPGS protein family was constructed by neighbor-joining using Geneious Prime 2019 software (www.geneious.com).

### Vector Construction and Transgenic Plant Production

The RNAi construct was made using the switchgrass *PvFPGS1* gene sequence (Pavir.Ib00114). A 462 bp sequence (Supplementary Figure S1) was amplified by PCR from switchgrass cultivar ‘Alamo’ using primer pair PvDFB-RNAi-F: 5′-AAGCAGGGGCATAAGGACA-3′ and PvDFB-RNAi-R: 5′-ATCGATTTGTTCAGGCTCAGC-3′. The target fragment was cloned into pCR8 entry vector and confirmed by sequencing. The target fragment was then sub-cloned into pANIC-8A RNAi-vector (Supplementary Figure S2) (Mann et al., 2012) to be driven under the maize ubiquitin 1 (*ZmUbi1*) promoter. Transgenic plants were produced using NFCX01 clonal of switchgrass ‘Alamo’ via *Agrobacterium*-mediated transformation (Xi et al., 2009).

### Greenhouse Plants

Plants were grown in greenhouse under the 16-h day/8-h night light at 28°C day/22°C night temperature.

### Field-Grown Plants and Experimental Design

The T_0_ generation of *PvFPGS1*-downregulated plants were used in field experiments. The plants included six independent transgenic lines and one non-transgenic control (wild type). The plants were transplanted onto a field on June 05, 2014. The field was located at the University of Tennessee Plant Sciences Unit of the East Tennessee Research and Education Center (ETREC). The field site was 24.2 m × 15.1 m. Three replicate plots for each transgenic and control lines were distributed throughout the field in a randomized complete block design (RCBD). Each transgenic and control replicate plot contained four vegetatively propagated clones of each line. Replicate lines were spaced 152 cm apart with 76 cm between the four clonal plants within every single replicate. The experimental plots were surrounded by non-transgenic border plants (Supplementary Figure S3).

### Field Maintenance

The field trial was conducted for three consecutive growing seasons, in which no environmental anomalies were observed. The soil fertility was in the range of switchgrass recommendations and no soil amendments were added. The plants were irrigated only for the first 2 months as needed after transplantation for establishment. Weeds were removed by tilling or hand. No herbicides were applied for the duration of the study. Following USDA APHIS BRS guidelines, plants were observed daily during the reproductive stage and emerging panicles were removed from all plants (transgenic, non-transgenic control and border plants) at R0-R1 developmental stage (Moore et al., 1991) by cutting the plant below the top node containing the inflorescence.

### Analysis of *PvFPGS1* Transcript Levels

Baseline expression of *FPGS1* in different tissues of switchgrass at the R1 growth stage was tested in greenhouse-grown plants using quantitative reverse transcription-polymerase chain reaction (qRT-PCR). Total RNA was extracted from 1st, 2nd, 3rd, and 4th internodes; 1st, 2nd, 3rd, and 4th nodes; leaf blade; leaf sheath; crown; inflorescence; and root of non-trangenic lines or from the leaf blade, leaf sheath, and 2nd internode of transgenic lines at the R1 growth stage. For field-grown plants, samples were collected from green plants in August of each growing season. All samples were collected at the same date and time of each year analyzed. Tillers at the R0 developmental stage were chosen at random from two plants within each replicate. Each tiller was excised below the top internode. The resulting top portion of the tiller with the two intact top leaves was flash-frozen in liquid nitrogen and stored at −80°C for qRT-PCR analysis. Total RNA was isolated from the frozen tissues using Spectrum^TM^ Plant Total RNA Kits (Sigma-Aldrich, St. Louis, MO, United States) following the manufacturer’s instructions. RNA quality was checked with Agilent Bioanalyzer 2100 (Agilent, Palo Alto, CA, United States) and quantified using Qubit^TM^RNA BR Assay Kit (Fisher Scientific, Santa Clara, CA, United States). Five micrograms of total RNA was treated with TURBO DNA-free^TM^ Kit (Invitrogen, Carlsbad, CA, United States) to remove any potential genomic DNA contaminants. Two micrograms of DNA-free total RNA was used for first-strand cDNA synthesis using SuperScript^TM^ III First-Strand Synthesis System (Invitrogen). qRT-PCR was performed with Power SYBR^TM^ Green PCR Master Mix (Applied Biosystems, Foster City, CA, United States) using ABI PRISM 7900 HT Sequence Detection System (Applied Biosystems). The primer pair used for the *PvFPGS1* transcript analysis was PvDFB-RNAi-qRT-F1: 5′-CAAAGAGCTTCGGAGTTGG-3′ and PvDFB-RNAi-qRT-R1: 5′-GGTAGGGGATCAGTACGATTGA-3′. Data were collected and analyzed using the SDS 2.2.1 software (Applied Biosystems). The relative transcript quantification was normalized by the levels of switchgrass ubiquitin 1 (*PvUbi1*) transcripts (Shen et al., 2009) using primer pair SWUbi_F304: 5′-TTCGTGGTGGCCAGTAAGC-3′ and SWUbi_R367: 5′-AGAGACCAGAAGACCCAGGTACAG-3′.

### Agronomic Performance

Growth measurements were recorded each December (end-of-season) of the three growing seasons in the field after all aboveground biomass was completely senesced. For tiller height, the tallest tiller from each individual plant was measured from soil level to the tip of the top leaf. For plant width, the circumference at the mid-section of each whole plant was measured. Tiller numbers were tallied for each plant. For biomass yield, whole aboveground senesced biomass was harvested. The biomass was oven-dried at 43°C for 96 h and weighed to determine total dry aboveground biomass. The dried biomass samples were chipped into 5–8 pieces prior to milling. The chipped samples were milled with a Wiley mill (Thomas Scientific, Model 4, Swedesboro, NJ, United States) through a 20-mesh screen (1.0 mm particle size). This milled biomass was used for cell wall characterization and bioconversion analyses.

### Cell Wall Characterization

Lignin content and composition were determined by pyrolysis molecular beam mass spectrometry (py-MBMS) using the NREL high-throughput method wherein soluble extractive and starch were removed from the biomass samples (Sykes et al., 2009; Decker et al., 2012). Lignin content was estimated from the relative intensities of the lignin precursor peaks. S/G lignin monomer ratio was determined by dividing the sum of the intensity of syringyl peaks by the sum of the intensity of guaiacyl peaks. Sugar release by enzymatic hydrolysis was determined using the NREL high-throughput method as previously described (Selig et al., 2010). Cell wall residues prepared by removing soluble extractives and starch were subjected to a hot water pretreatment (180°C for 17.5 min) followed by a 72-h incubation at 40°C with hydrolyzing enzymes. Glucose and xylose release were determined by colorimetric assays, and total sugar release is the sum of glucose + xylose released (Studer et al., 2009).

### Ethanol Yield

Ethanol yield was determined by separate hydrolysis and fermentation (SHF) as described previously (Dumitrache et al., 2017). Biomass samples were incubated at 50°C for 5 days with hydrolyzing enzymes. The resulting sugars were fermented at 35°C for 72 h with *Saccharomyces cerevisiae* D5α (ATCC 200062). Ethanol yield was determined at the endpoint by HPLC quantification (Bio-Rad, Hercules, CA, United States).

### RNA-Seq Analysis

The whole tillers and internode of the greenhouse-grown plants at the R1 developmental stage were harvested for RNA-seq analysis as described previously (Rao et al., 2019). RNA-seq was conducted at Joint Genome Institute (JGI) using Illumina TruSeq technology. Four biological replicates for each sample group were included. For each sample of the *PvFPGS1*-downregulated and control lines, a total of 40–50 million paired-end (PE) reads of 150 bp was generated. Paired-end Illumina reads after filtering and trimming treatment were mapped to the Switchgrass genome *Panicum virgatum* v3.1 using HISAT2 (Kim et al., 2015) with default parameters. The selected genes were annotated with switchgrass genome v5.1^1^. Genes whose expression was different from the control were selected through comparison between each transgenic line and its control, using differential analysis software such as DESeq (Anders and Huber, 2010) with default settings of adjusted *P*-value < 0.05. Genes were annotated against Arabidopsis, rice and other model species using blast search.

### Rust Disease Evaluation

Plants were evaluated for rust disease caused by *Puccinia novopanici* (formerly known as *Puccinia emaculata*) infection. Disease severity was assessed at weekly time points during the second (2015) and third (2016) growing seasons between July and August as described by Baxter et al. (2018). Two plants within each replicate were selected at random. A single tiller from each plant was tagged and all leaves on the selected tillers were examined for rust severity. The coverage of the top leaf surface with rust uredia was visually evaluated using the following scale: 0 = 0%, 1 ≤ 5%, 2 ≤ 10%, 3 ≤ 25%, 4 ≤ 40%, 5 ≤ 55%, 6 ≤ 70%, and 7 ≤ 100%. Because of the severity of the rust, the whole field was treated with fungicide in late August of each growing season. All data reported were collected before fungicide treatments. Fungicides used included “Quilt” (Syngenta Canada Inc., Guelph, ON, Canada) at a rate of 0.21 ml/m^2^, and “Heritage” (Syngenta Crop Protection, Greensboro, NC, United States) at a rate of 20 ml/m^2^.

### Statistics

Means were analyzed with one-way ANOVA using Fisher’s least significant difference method in SAS version 9.4 (SAS Institute Inc., Cary, NC, United States). Differences were considered statistically significant where *P*-values were less than 0.05.

## Results

### Identification of PvFPGS Homologs

**FIGURE 1:**
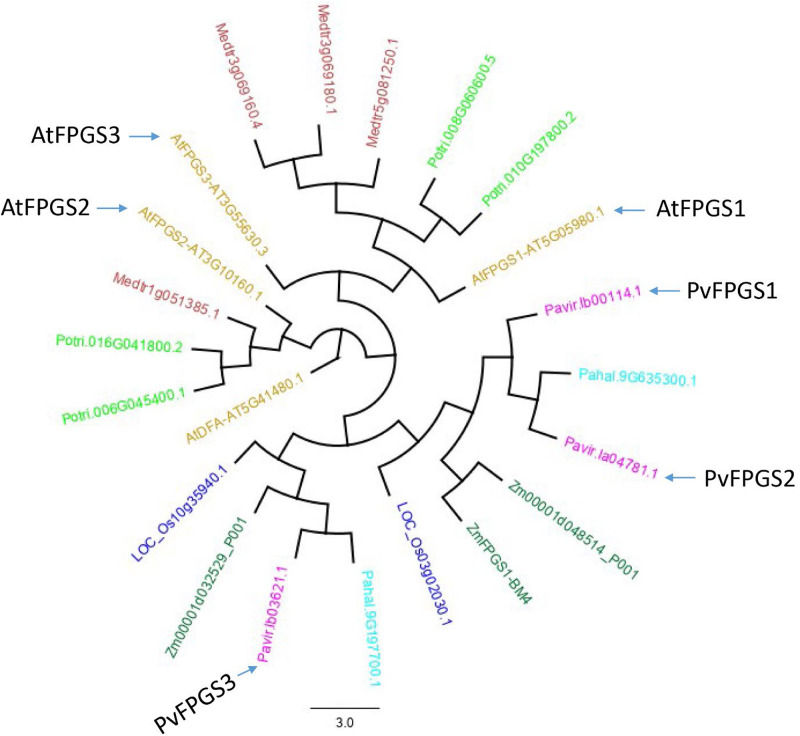
The gene tree of the FPGS protein family. Various members are shown by plant species: *Arabidopsis thaliana* (gold), *Populus trichocarpa* (light green), *Medicago truncatula* (brown), *Oryza sativa* (blue), *Zea mays* (green), *Panicum hallii* (light blue), and *Panicum virgatum* (pink) from Phytozome 12 (https://phytozome.jgi.doe.gov/) showing relationship based on amino acid sequences. The phylogenetic analysis shows that switchgrass genome has three isoforms of FPGS: *PvFPGS1* (Pavir.Ib00114.1), *PvFPGS2* (Pavir.Ia04781.1), and *PvFPGS3* (Pavir.Ib03621.1). Based on this whole genome level comparison, the relationship of FPGSs between monocot and dicot is not as straightforward. However, among all three FPGSs in Arabidopsis and switchgrass, AtFPGS1 is still closest to PvFPGS1. In this graph, AtDFA encodes a dihydrofolate synthetase, homologous to FPGS but with different functions and serve as outgroup for this tree.

Switchgrass FPGS (PvFPGS) was initially identified using the FPGS amino acid sequences from *Arabidopsis thaliana* (At5g05980). Analysis of the sequences showed that switchgrass assembly v1.1 has only three isoforms of FPGS: Pavir.Ib00114.1, Pavir.Ib03621.1, and Pavir.Ia04781.1. A phylogenetic tree was constructed using all three isoforms of switchgrass and Arabidopsis as well as FPGS variants from several other species. These species, *Populus trichocarpa*, *Medicago truncatula*, *Oryza sativa*, *Zea mays*, and *Panicum hallii* from Phytozome 12, served as reference to elucidate homologous relationship among different members of FPGSs. Based on homology analysis, Pavir.Ib00114 was identified and named PvFPGS1 (Figure 1). There are two other isoforms of FPGS in switchgrass genome: *PvFPGS2* (Pavir.Ia04781.1) and *PvFPGS3* (Pavir.Ib03621.1). Based on this whole genome level phylogeny analysis of FPGSs, the relationship of FPGSs between monocot and dicot is not as straightforward as one to one, especially AtFPGS3 had no isologs in other species. However, among all three FPGSs between Arabidopsis and switchgrass, AtFPGS1 is still closest to PvFPGS1 with an identity of 59.4%. Moreover, *PvFPGS1* is isolog of maize *brown midrib4* (*bm4*) which encode a functional FPGS and its loss-of-function leads to lower lignin content (Li et al., 2015).

### Expression Patterns of *PvFPGS1* in Switchgrass

**FIGURE 2:**
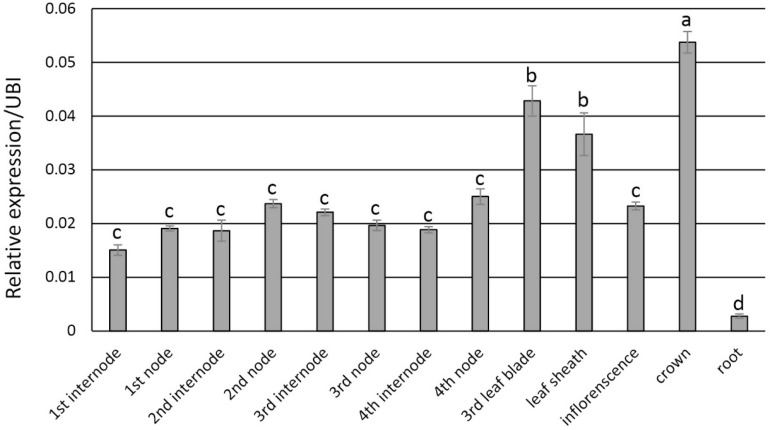
Expression patterns of *PvFPGS1* in different plant tissues as determined by qRT-PCR. Plant samples for RNA extraction used in the qRT-PCR experiments were collected at R1 (reproductive stage 1) developmental stage. The relative levels of transcripts were normalized to the switchgrass ubiquitin 1 gene expression (UBI). Bars represent mean values of three biological replicates ± standard error. Means were compared by a one-way ANOVA and letter groupings were obtained using Fisher’s least significant difference method. Bars with different letters are significantly different at the 5% level.

**FIGURE 3:**
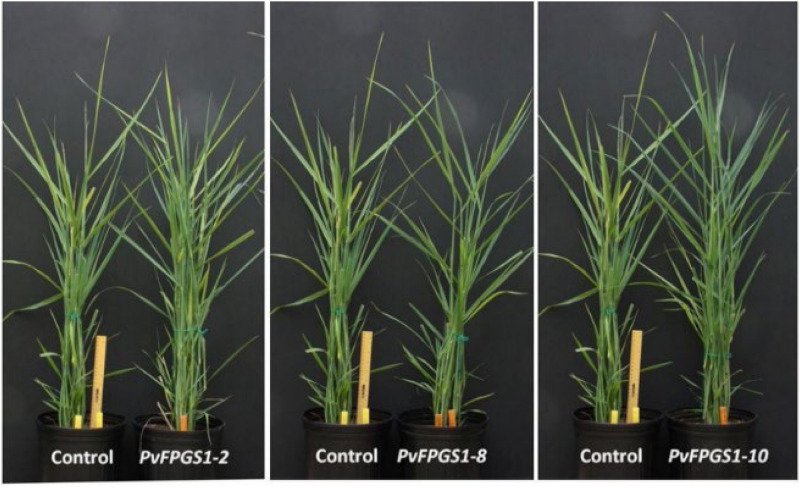
Representative *PvFPGS1*-RNAi transgenic and non-transgenic control lines grown under greenhouse conditions at 3 months old.

Transcript abundance via qRT-PCR analysis indicated that *PvFPGS1* in non-transgenic plants is expressed in stems, leaves, crown, inflorescences, and roots at the R1 developmental stage. The level of *PvFPGS1* expression was highest in the crown and leaves, and lowest in the root (Figure 2). *PvFPGS1* transcripts were detected in all the tissue types tested. In the transgenic plants, depending on the transgenic event, *PvFPGS1* transcript abundance was reduced by 23–82% in the joint leaf blade, leaf sheath, and 2nd internode tissues of the RNAi-transgenic lines (Supplementary Figure S4). Regardless of the level of reduction in *PvFPGS1* expression, the growth and phenotype of transgenic lines was not apparently different from non-transgenic controls under greenhouse conditions (Figure 3).

### Field Experiments

**FIGURE 4:**
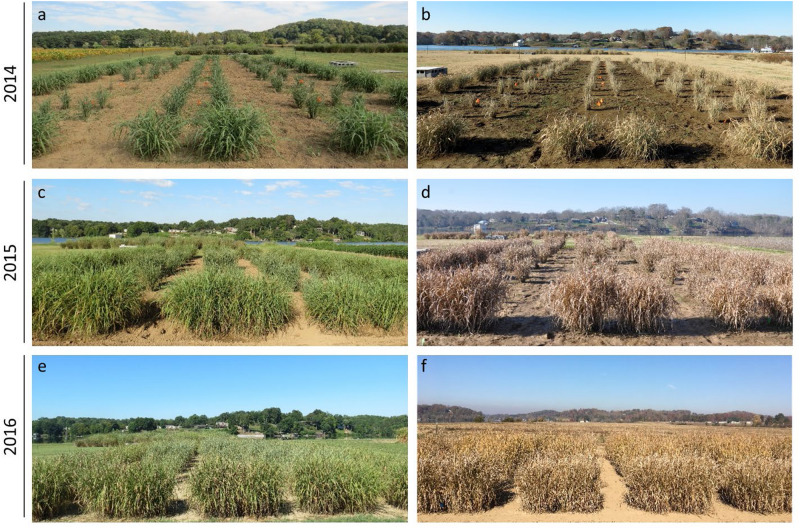
Photos of *FPGS1*-downregulated switchgrass in the field. **(a)** October 01, 2014; **(b)** November 21, 2014; **(c)** August 26, 2015; **(d)** December 08, 2015; **(e)** August 16, 2016; **(f)** November 17, 2016.

From greenhouse studies, we selected six independent transgenic lines for the field experiment, which had a range of decreased *PvFPGS1* expression. The plants included the transgenic lines T2 (decreased expression by 71%), T8 (decreased expression by 72%), T10 (decreased expression by 73%), T12 (decreased expression by 82%), T32 (decreased expression by 67%), T115 (decreased expression by 63%), and one non-transgenic control. The field study was conducted for three consecutive growing seasons (2014–2016) (Figure 4).

### *PvFPGS1* Gene Expression Under Field Conditions

**FIGURE 5:**
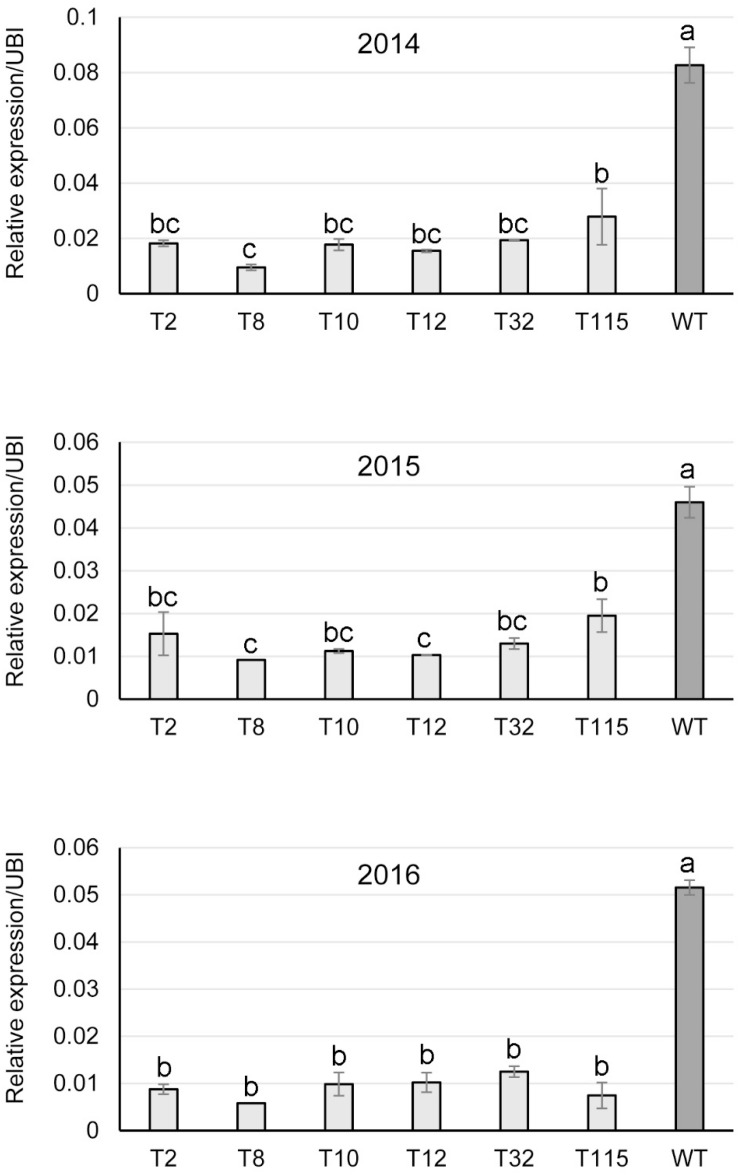
Relative transcript levels of *PvFPGS1* in RNAi-transgenic lines as determined by qRT-PCR. Samples were collected in year one (2014), year two (2015) and year 3 (2016) of the field trial. WT: non-transgenic control. The relative levels of transcripts were normalized to the switchgrass ubiquitin 1 gene expression (UBI). Bars represent mean values of three biological replicates ± standard error, with the exception of line T8 in 2015 and 2016, which is from one surviving plant during the two growing seasons. Means within each year were compared by a one-way ANOVA and letter groupings were obtained using Fisher’s least significant difference method. Bars with different letters are significantly different at the 5% level.

Transgene expression in the field-grown *PvFPGS1*-downregulated switchgrass lines was studied by qRT-PCR on sequentially harvested tissue over the course of the field study. *PvFPGS1* transcript levels were reduced in the transgenic lines for field year one (2014), year two (2015), and year three (2016) compared to the control. The *PvFPGS1* expression was lowest in transgenic line T8 with 80–89% decrease in transcript level compared to the control in years one, two, and three. The decrease in *PvFPGS1* transcript level was followed by transgenic lines T2 (67–83%), T10 (76–81%), T12 (78–81%), T32 (72–77%), and T115 (by 58–86%) relative to the control during the three growing seasons (Figure 5).

### Agronomic Performance

For each growing season, the following end-of-year growth characteristics were assessed: tiller height, plant width, tiller number, and aboveground dry biomass. Biomass production of transgenic lines T10, T12, and T32 was comparable to that of the control in the first year (2014), while lines T2 (decreased biomass by 45%), T8 (decreased biomass by 81%), and T115 (decreased biomass by 62%) exhibited yield reduction. The yield reductions in these lines were congruent with decreased tiller height, plant width, and tiller number by up to 56%. In the second year (2015), the transgenic lines showed similar biomass yield and growth traits to that of the control with the exception of line T8, in which only one plant (out of 12 plants) survived the first field winter. The sole T8 survival had decreased biomass by 95% accompanied by up to 69% decrease in tiller height, plant width, and tiller number compared to the control. Similar to the second year, transgenic lines did not differ from the control in biomass production in the third year (2016), except for the T8 surviving plant which had a 99% decrease in biomass yield accompanied by up to 94% reduction in tiller height, plant width, and tiller number relative to the control (Table 1).

**TABLE 1:** Morphology and dry weight yield of *PvFPGS1*-downregulated lines at the first (2014), second (2015), and third (2016) growing seasons per plant.

Year	Line	Tiller height (cm)	Plant width (cm)	Tiller number	Dry weight yield (g/plant)
2014	T2	**44.5 ± 14.3^c^**	**36.7 ± 12.1^b^**	**31.0 ± 2.6^b^**	**36.0 ± 2.4^b^**
	T8	**56.0 ± 0.6^bc^**	**41.7 ± 2.4^b^**	**28.8 ± 5.7^b^**	**12.3 ± 2.4^c^**
	T10	80.0 ± 1.7^a^	78.5 ± 1.5^a^	58.4 ± 7.4^a^	59.7 ± 2.9^a^
	T12	82.3 ± 2.3^a^	76.2 ± 6.5^a^	54.8 ± 2.4^a^	67.3 ± 7.5^a^
	T32	86.6 ± 2.0^a^	91.7 ± 1.8^a^	58.1 ± 3.8^a^	83.7 ± 3.4^a^
	T115	69.7 ± 10.0^ab^	**46.1 ± 12.1^b^**	**22.4 ± 8.3^b^**	**25.0 ± 8.5^b^**
	WT	78.4 ± 0.8^a^	83.4 ± 5.1^a^	60.4 ± 3.1^a^	65.8 ± 6.3^a^
2015	T2	101.8 ± 1.3^ab^	106.4 ± 14.6^a^	80.4 ± 16.1^a^	178.6 ± 36.3^a^
	T8*	**63.5 ± 0.0^c^**	**35.6 ± 0.0^b^**	**34.0 ± 0.0^b^**	**10.0 ± 0.0^b^**
	T10	104.7 ± 6.6^ab^	114.7 ± 7.4^a^	118.1 ± 12.7^a^	204.2 ± 20.4^a^
	T12	116.2 ± 12.0^a^	128.9 ± 22.3^a^	110.3 ± 20.8^a^	311.7 ± 114.8^a^
	T32	107.1 ± 6.7^a^	133.4 ± 8.6^a^	113.8 ± 6.5^a^	308.9 ± 64.0^a^
	T115	88.9 ± 2.9^b^	99.1 ± 22.6^a^	77.1 ± 22.6^a^	142.5 ± 48.5^a^
	WT	100.4 ± 3.5^ab^	116.3 ± 1.0^a^	96.4 ± 13.2^a^	206.1 ± 11.5^a^
2016	T2	**127.7 ± 9.0^b^**	189.2 ± 56.5^a^	96.9 ± 7.8^a^	446.8 ± 98.0^a^
	T8*	**34.3 ± 0.0^c^**	**15.2 ± 0.0^b^**	**11.0 ± 0.0^b^**	**4.5 ± 0.0^b^**
	T10	152.5 ± 4.5^a^	208.1 ± 8.8^a^	121.5 ± 6.0^a^	593.3 ± 68.8^a^
	T12	162.8 ± 6.3^a^	220.8 ± 34.7^a^	125.4 ± 21.1^a^	741.3 ± 211.5^a^
	T32	153.1 ± 3.1^a^	226.6 ± 5.9^a^	135.7 ± 5.5^a^	782.4 ± 38.5^a^
	T115	**136.3 ± 4.2^b^**	185.0 ± 29.5^a^	97.2 ± 25.6^a^	378.5 ± 65.4^a^
	WT	161.8 ± 5.7^a^	242.7 ± 13.1^a^	119.5 ± 12.5^a^	752.4 ± 68.8^a^

Values are the mean of three biological replicates for each transgenic line (T2, T8, T10, T12, T32, and T115) and wild-type control (WT) ± standard error, with the exception of line T8 in 2015 and 2016, which is from one surviving plant during the two growing seasons. Means within each year were compared by a one-way ANOVA and letter groupings were obtained using Fisher’s least significant difference method. Values followed by different letters are significantly different at the 5% level. Bold values are significantly different from controls. *Only one surviving plant.

### Lignin Content and Composition

Cell wall lignin content and the S/G ratio were measured for aboveground biomass harvested at end-of-season of each year by pyrolysis molecular beam mass spectrometry (py-MBMS). Decrease in lignin content (14% reduction) and S/G ratio (18% reduction) were only observed in line T8 relative to the control in year one (2014). There were no significant differences in lignin content or S/G ratio between all the other transgenic lines and the control lines at the first (2014), second (2015), and third (2016) growing seasons (Table 2).

**TABLE 2:** Lignin content, S/G ratios, and sugar release of *PvFPGS1*-downregulated lines at the first (2014), second (2015), and third (2016) growing seasons.

Year	Line	Lignin content (% CWR)	*S*/*G* ratio	Glucose release (mg/g CWR)	Xylose release (mg/g CWR)	Total sugar release (g/g CWR)
2014	T2	20.4 ± 0.3^b^	0.59 ± 0.01^ab^	0.219 ± 0.00^a^	0.178 ± 0.00^b^	0.396 ± 0.00^b^
	T8	**18.3 ± 0.4^c^**	**0.49 ± 0.02^c^**	0.205 ± 0.00^a^	**0.154 ± 0.00^c^**	**0.359 ± 0.01^c^**
	T10	20.8 ± 0.2^b^	0.56 ± 0.01^b^	0.228 ± 0.00^a^	0.183 ± 0.00^b^	0.410 ± 0.00^ab^
	T12	21.2 ± 0.2^ab^	0.58 ± 0.02^ab^	0.236 ± 0.01^a^	**0.195 ± 0.00^a^**	**0.430 ± 0.01^a^**
	T32	22.0 ± 0.2^a^	0.61 ± 0.01^a^	0.216 ± 0.01^a^	0.185 ± 0.01^ab^	0.401 ± 0.01^b^
	T115	20.4 ± 0.4^b^	0.57 ± 0.02^ab^	0.238 ± 0.01^a^	0.177 ± 0.00^b^	0.414 ± 0.02^ab^
	WT	21.1 ± 0.3^ab^	0.59 ± 0.01^ab^	0.215 ± 0.00^a^	0.177 ± 0.00^b^	0.392 ± 0.00^b^
2015	T2	20.8 ± 0.2^a^	0.68 ± 0.02^a^	0.190 ± 0.00^c^	**0.183 ± 0.01^c^**	**0.374 ± 0.00^c^**
	T8*	20.8 ± 0.0^a^	0.64 ± 0.00^a^	0.229 ± 0.00^a^	0.205 ± 0.00^ab^	0.434 ± 0.00^a^
	T10	21.0 ± 0.1^a^	0.67 ± 0.00^a^	0.216 ± 0.01^ab^	0.211 ± 0.01^a^	0.427 ± 0.02^a^
	T12	21.5 ± 0.5^a^	0.65 ± 0.02^a^	0.205 ± 0.00^bc^	0.209 ± 0.01^a^	0.414 ± 0.00^ab^
	T32	22.3 ± 0.5^a^	0.70 ± 0.01^a^	0.201 ± 0.01^bc^	0.209 ± 0.00^a^	0.409 ± 0.01^ab^
	T115	21.3 ± 0.4^a^	0.71 ± 0.02^a^	0.202 ± 0.00^bc^	0.191 ± 0.01^bc^	0.393 ± 0.01^bc^
	WT	21.5 ± 0.4^a^	0.68 ± 0.02^a^	0.205 ± 0.01^bc^	0.209 ± 0.01^a^	0.414 ± 0.01^ab^
2016	T2	22.8 ± 0.3^a^	0.78 ± 0.02^a^	0.151 ± 0.01^a^	0.167 ± 0.00^ab^	0.318 ± 0.01^a^
	T8*	ND	ND	ND	ND	ND
	T10	22.3 ± 0.4^a^	0.75 ± 0.02^a^	0.155 ± 0.00^a^	**0.174 ± 0.00^a^**	0.329 ± 0.00^a^
	T12	22.7 ± 0.1^a^	0.71 ± 0.01^a^	0.149 ± 0.01^a^	**0.172 ± 0.00^a^**	0.321 ± 0.01^a^
	T32	23.0 ± 0.2^a^	0.77 ± 0.02^a^	0.140 ± 0.00^a^	0.168 ± 0.00^ab^	0.308 ± 0.00^a^
	T115	22.7 ± 0.6^a^	0.79 ± 0.05^a^	0.142 ± 0.00^a^	0.162 ± 0.00^b^	0.304 ± 0.00^a^
	WT	23.4 ± 0.2^a^	0.78 ± 0.01^a^	0.142 ± 0.01^a^	0.163 ± 0.01^b^	0.306 ± 0.01^a^

*Values are the mean of the biological replicates (n = 3) for each transgenic line (T2, T8, T10, T12, T32, and T115) and wild-type control (WT) ± standard error, with the exception of line T8 in 2015 and 2016, which is from one surviving plant during the two growing seasons. Means within each year were compared by a one-way ANOVA and letter groupings were obtained using Fisher’s least significant difference method. Values followed by different letters are significantly different at the 5% level. Bold values are significantly different from controls. ^∗^Only one surviving plant. ND, not done due to unavailability of material needed for the analysis. CWR: cell wall residue.*

### Sugar Release Efficiency

Enzymatic hydrolysis was used to determine the sugar release for aboveground biomass harvested at end of each season. In year one (2014), line T8 exhibited a decrease in xylose (13%) and total sugar (8%) release relative to the control, whereas line T12 had a 10% higher xylose and total sugar release than control. In year two (2015), a decrease in xylose (12%) and total sugar (10%) release was only observed in line T2. In year three (2016), there was an increase in xylose release in lines T10 (7%) and T12 (5%). There were no significant differences in sugar release between the other transgenic and the control lines at the first (2014), second (2015), and third (2016) growing seasons (Table 2).

### Ethanol Yield

**FIGURE 6:**
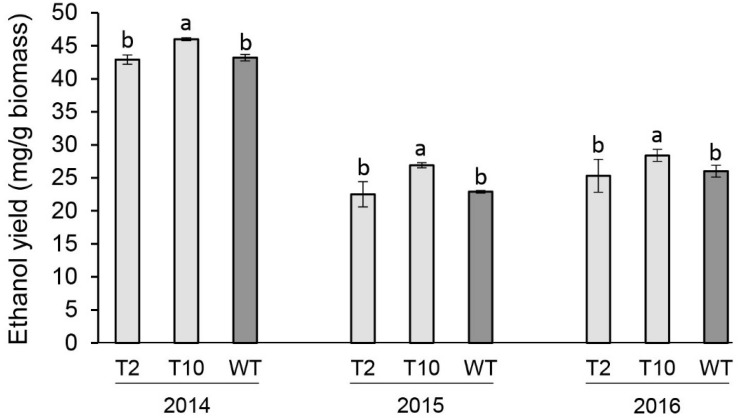
Ethanol yields of *FPGS1*-downregulated switchgrass in the first (2014), second (2015), and third (2016) growing seasons. Bars are the mean value of three biological replicates for each transgenic line (T2 and T10) and wild-type control (WT) ± standard error. Means within each year were compared by a one-way ANOVA and letter groupings were obtained using Fisher’s least significant difference method. Bars with different letters are significantly different at the 5% level.

The fermentation potential of aboveground biomass harvested at end-of-season of each year was determined by separate hydrolysis and fermentation (SHF). Lines T2 and T10 with moderate levels of the decreased *PvFPGS1* expression were selected to evaluate bioconversion efficiency. Line T10 showed an increase in ethanol yield in year one (2014) by 7%, year two (2015) by 18%, and year three (2016) by 9% relative to the control. There were no differences in ethanol yield between line T2 and the control at the three growing seasons (Figure 6).

### RNA-Seq Analysis

Transcriptome analysis by RNA-seq was conducted to identify underlying pathways through which modification may further improve biofuel production. In both tiller and internode, the number of putative genes of different expression in the *PvFPGS1*-downregulated line T10 compared with control was similar, with 148 higher and 145 lower in tiller while 134 higher and 167 lower in the internode (Supplementary Table S1). FPGS1 has two subtypes presented in the annotated switchgrass genome, one in K genome and one in the N genome, each was named based on their subgenome, namely FPGS1K (Pavir.9KG559800 V5.1, originally Pavir.Ib00114.1 in V1.1), and FPGS1N (Pavir.9NG789900 V5.1, originally Pavir.Ia04781.1 in V1.1). Sequence identity between these two subtypes is 96.7%, and both were significantly reduced to 20–30% of the control levels, indicating these target genes were successfully downregulated in line T10. Reads for switchgrass FPGS2 is not well mapped in this RNA-seq data and its expression pattern will be worth looking at in future study. In total, there were 565 genes that were differentially expressed in line T10. Based on gene annotation, 38 differentially expressed genes were putatively involved in activities related to plant cell wall (Supplementary Table S2). Among them, in addition to the FPGS1 target genes, seven genes encode enzymes in the flavonoid and aromatic amino acids synthesis pathways which directly interact with the phenylpropanoid pathway, the upstream of lignin biosynthesis pathway. These genes include *chalcone synthase* (*CHS*) (Pavir.8NG107400 and Pavir.8KG261900), *chalcone isomerase* (Pavir.9NG037000), *chorismate mutase* (Pavir.1KG086400), *isochorismate synthase* (Pavir.2KG294000), *anthocyanidin reductase* (Pavir.7NG368500), and *isoflavone 7-O-glucosyltransferase* (Pavir.9NG095000). Peroxidase and laccase are enzymes involved in lignin polymerization and deposition (Tobimatsu and Schuetz, 2019). Three peroxidase (Pavir.2KG513700, Pavir.2NG638600, and Pavir.7NG321900) and three laccase genes (Pavir.5KG623300, Pavir.5NG585100, and Pavir.5KG613400) are among the selected genes. One gene potentially involved in monolignol metabolism, monolignol beta-glucoside homolog without catalytic acid/base, also present in the selected gene list (Pavir.7NG350700). Ten genes involved in different steps of some type of sugar and wall polysaccharide synthesis and modification are also among the selected genes. At the upstream of the pathway, two genes involved in myo-inositol metabolism have reduced expression in line T10, *myo-inositol-1-phosphate synthase* (Pavir.9KG643000) and *myo-inositol 2-dehydrogenase* (Pavir.2NG256100). Following these steps, four UDP nucleotide sugar metabolism enzymes are also among the mostly downregulated gene list: *UDP-arabinose 4-epimerase* (Pavir.9KG554500), *UDP-arabinopyranose mutase 1 related* (Pavir.9NG214400), *UDP-glycosyltransferase 73B4* (Pavir.7NG278900), *UDP-glucuronic acid decarboxylase* (Pavir.9KG487800). Genes involved in pectin and hemicellulose also are among genes of changed expression in line T10. Among them are *polygalacturonate 4-alpha-galacturonosyltransferase* (*GAUT4*) (Pavir.2KG409000), *cellulose synthase* (*CESA8*) (Pavir.2KG167600), *licheninase/mixed linkage beta-glucanase* (Pavir.3NG120500); three glycotransferase (Pavir.5NG133600, Pavir.1KG262700, and Pavir.4KG337800) are all downregulated in line T10; the last group of genes on the list that are directly involved in cell wall structure are five wall associated proteins: four hydroxyproline-rich glycoprotein family protein (Pavir.2KG047700, Pavir.2KG047500, Pavir.3KG332300, and Pavir.9NG522300); the other one is *wall-associated kinase 2* (Pavir.7NG318000). One interesting gene that is downregulated in the internode of line T10 encodes an EamA-like transporter family member (Pavir.5KG129300), homology to nodulin (MtN21) and walls are thin 1 (WAT1), which is a tonoplast-localized protein required for secondary wall formation in fibers (Ranocha et al., 2010).

### Disease Susceptibility

All plants were evaluated for rust disease severity during the second (2015) and third (2016) growing seasons. Rust disease was detected in late July and infection was advanced largely through late August for both growing seasons. In order to maintain the field for downstream analyses, the field was sprayed with fungicides. Disease severity of rust infection was rated weekly prior to fungicide treatments based on the percentage of the leaf area coverage with rust uredia. The disease severity ranged from 0 to 9% during the second (2015) and 4–18% during the third (2016) growing seasons. There were no significant differences in rust susceptibility between transgenic and control lines for both growing seasons (Supplementary Figures S5, S6).

## Discussion

A clear understanding of cell wall enzymology is needed to engineer reduced recalcitrance in bioenergy crops. We previously reported that the loss of function of Arabidopsis FPGS1 resulted in lignin reduction and improved saccharification efficiency in Arabidopsis (Srivastava et al., 2015; Xie et al., 2019). Given the link between the FPGS and lignin biosynthetic pathways, our findings gave rise to the hypothesis that FPGS plays a functional role in reducing recalcitrance in switchgrass, a leading lignocellulosic bioenergy feedstock. The present study describes identification of switchgrass *FPGS* gene (*PvFPGS1*). Our study strengthens the FPGS-recalcitrance hypothesis.

In Arabidopsis, there are three genes that each encode FPGS isoforms: FPGS1, FPGS2, and FPGS3 (Ravanel et al., 2001). The homologous sequence in switchgrass discovered using AtFPGS1 was named PvFPGS1. At whole genome level, PvFPGS1 has two subtypes, each belong to the N and K subgenome, they show similarity closer to AtFPGS1 and AtFPGS3, farther from AtFPGS2.

Endogenous expression of *PvFPGS1* transcripts was highest in the crown of plants. Transcript abundance decreased sequentially in leaves, internodes, inflorescences, and roots. The expression profile of *PvFPGS1* is slightly different from Arabidopsis as the expression profile of Arabidopsis *AtFPGS1* showed the highest expression in stems compared to other tissues (Srivastava et al., 2015). Secondary cell walls consisted of high amount of lignin provide much of the rigidity in stem tissue, in contrast to the flexible organs such as roots. Consistently, expression of secondary cell wall-related genes has been shown to be higher in stems (Zhao and Bartley, 2014; Mazarei et al., 2018; Rao et al., 2019). Noteworthy, other than inflorescence, the tissues of high *PvFPGS1* expression in switchgrass are all significantly lignified (Crowe et al., 2017).

All transgenic *PvFPGS1*-downregulated lines had reduced targeted transcript levels over the three growing seasons of the field trial. These results confirm that the *PvFPGS1*-downregulated lines grown under field conditions sustained the reduction in *PvFPGS1* transcript levels compared to the control. For most of transgenic lines, plant growth was comparable to the control plants. However, in the field, the transgenic *PvFPGS1*-downregulating line T8, which had the highest decrease in *PvFPGS1* transcript levels (up to 89%) among lines either did not survive or had up to 99% reduction in biomass production over the control. The reduced biomass yield was accompanied with significant decrease in tiller height, plant width, and tiller number relative to the control. Yet, the transgenic line T8, which had the highest reduction in *PvFPGS1* expression had normal growth under greenhouse conditions. These observations that the transgenic lines with normal plant growth and development under greenhouse conditions could not survive or had substantial biomass loss under field conditions emphasize the importance of performing field studies. Likewise, an association between levels of transgene expression and biomass production for switchgrass grown under field conditions has been shown for transgenic *PvMYB4*-overexpressing lines, where the transgenic lines with higher expression levels of *PvMYB4* did not survive and/or had substantial reduction in biomass relative to control when grown under field conditions (Baxter et al., 2015). Yet, transgenic lines with low to moderate decreases in *PvFPGS1* expression levels produced biomass yield comparable to that of the controls. These observations highlight the significance of an optimized level of gene expression in transgenic plants.

Lignin content and S/G ratios were not significantly different in low-to-moderate downregulated *PvFPGS1* transgenic lines. Similarly, these transgenic lines showed similar sugar release efficiency compared with the control lines. Although our results are in contrast to Arabidopsis FPGS1 findings in which a loss of function was associated with reduced lignin content, coupled with an increase in sugar release efficiency (Srivastava et al., 2015; Xie et al., 2019), they used homozygous null-mutants in their studies. Similar observations were shown in maize where disruption in a gene encoding the isolog of PvFPGS1 resulted in low lignin (Li et al., 2015). We observed similar reductions in lignin in the T8 line where expression of FPGS1 was reduced to 80%. Cell wall lignin is one of the barriers to lignocellulosic biofuel production limiting digestion of cellulose into fermentable sugars. The manipulation of cell walls to decrease lignin content has been shown to improve bioconversion efficiency (Chen and Dixon, 2007; Baxter et al., 2014, 2015; Bonawitz et al., 2014; Eudes et al., 2014; Wilkerson et al., 2014; Hu et al., 2018). Since we did not find any noticeable difference in lignin content and composition in the selected *PvFPGS1*-downregulated lines, it is possible that the difference is very subtle and it is noticeable when we significantly disrupt the FPGS gene and associated supplies to the methylation pathway. This may also explain why there were not significant differences in sugar release efficiency between the selected *PvFPGS1*-downregulated lines and the controls.

Nevertheless, the *PvFPGS1*-downregulated line T10 produced up to 18% more ethanol than controls over the course of three field seasons. These observations may suggest that *PvFPGS1* expression is associated with improved biofuel production. Transcriptome data provides some clues to the increased ethanol production in the transgenic line T10. In grass plant, lignin can be synthesized from both phenylalanine and tyrosine and it is tightly linked to other secondary metabolite pathways, and tightly regulated through metabolites flux (Recent review by Vanholme et al., 2019). The secondary metabolites that derived from the same upper biochemical pathways but branched off to produce different type of flavonoids including anthocyanidin^2,3^. Genes in several steps leading to the synthesis of anthocyanidin show affected expression in line T10 indicating that this metabolite pathway could be affected. Work has shown that disturbance in flavone synthase II in rice (Lam et al., 2017) and CHS in maize (Eloy et al., 2017) have changed lignin content and digestibility. Increased level of *CHS* in line T10 with increased ethanol production is in agreement with this evidence. Even though the lignin content was not significantly different in line T10, decreased transcript levels in several laccases and peroxidases hint that the level of polymerization of lignin could be reduced, which could contribute to its increased ethanol production.

Furthermore, among the enzymes involved in wall simple sugar to polysaccharide synthesis and wall modifications, a number of the genes show affected expression levels in line T10. Wall polysaccharide formed a network of cellulosic, hemicellulosic, and pectic polysaccharides and protein (Bashline et al., 2014) and changes are dynamic. Although not all expression changes will have consequential effects as the plants maintain normal growth and have similar sugar release, however, changes in some steps have led accumulated changes that have contributed to the increased ethanol productions in line T10. In rice, mutants of a putative glycosyltransferase in a grass-specific subfamily of GT61, are deficient in ferulic acid, coumaric acid, and aromatic compounds and exhibit an increased saccharification efficiency (Chiniquy et al., 2012). It is worth noting that one of the genes annotated as glycosyltransferase family 61 protein among the selected gene list. Lower expression of this gene could indirectly affect the level of these small metabolites and hence could contribute to the higher level of ethanol in line T10. The other gene that could be of significant effect is gene encoding licheninase/mixed linkage beta-glucanase. This enzyme could cause release of smaller oligosaccharides (DP < 6) from graminaceous hemicelluloses (Yoshida and Komae, 2006). Some smaller oligosaccharides were shown to be recalcitrant to fermentation (Jonathan et al., 2017). In the line T10, expression level of licheninase is reduced and thus could add another factor that potentially contribute to its higher ethanol yield.

Besides, phenolic composition of switchgrass has been shown to be another important factor affecting recalcitrance (Tschaplinski et al., 2012; Yee et al., 2012; Shen et al., 2013; Baxter et al., 2015). Studies with *PvCOMT*-downregulated switchgrass plants had higher levels of certain phenolic compounds which inhibited the microbial fermentation (Tschaplinski et al., 2012; Yee et al., 2012). In contrast, *PvMYB4*-overexpressing switchgrass plants grown in the greenhouse and field had lower amounts of phenolic compounds that inhibit microbial fermentation (Shen et al., 2013; Baxter et al., 2015). Interestingly, one *PvMYB4*-overexpressing line showed no increase in sugar release efficiency but still resulted in higher levels of ethanol production when grown in the field (Baxter et al., 2015). These studies suggest that there are factors other than lignin, e.g., phenolic compounds, that play roles in improved biofuel production observed in transgenic switchgrass overexpressing *PvMYB4* (Shen et al., 2013; Baxter et al., 2015). Given that C1 metabolism pathway is involved in synthesis of variety of polymers and plant secondary metabolites, it is tempting to speculate that the increased ethanol production in the *PvFPGS1*-downregulated line may be caused by changes in phenolic fermentation inhibitors. Elucidating of this mechanism will be the subject of future study.

Successful establishment and sustainability of bioenergy feedstocks are key factors for production of fuel from biomass (Stewart and Cromey, 2011). Performing field studies of transgenic plants is crucial to examine consequences of their genetic modifications on plant defenses. Of particular significance is the rust disease caused by fungal pathogen *P. novopanici* that is identified as potentially damaging to switchgrass fields (Uppalapati et al., 2013). During the last two growing seasons, rust severity in *PvFPGS1*-downregulated lines was not different from that of control plants. Levels of *PvFPGS1* expression appears to not be a factor in switchgrass rust disease.

In conclusion, we have shown that genetic manipulation of *PvFPGS1* could lead to improved biofuel production without negatively impacting plant growth and biomass yield. These results provide further insights into the effect of knockdown expression of *PvFPGS1* on improving biofuel production in switchgrass. As of interest is to use *PvFPGS1* in complementation studies of the Arabidopsis and maize mutants with loss of function of native FPGS orthologs. This could further elucidate functionality of FPGS and provide information on potential strategies to enhance productivity in bioenergy crops. Further research on the FPGS genes enhance understanding of the factors associated with reducing recalcitrance. Our study provides a starting point for a more rigorous exploration of the role of *PvFPGS1* in the bioenergy field.

## Data Availability Statement

The original contributions presented in the study are included in the article/Supplementary Material, further inquiries can be directed to the corresponding authors.

## Author Contributions

MM designed the experiments, participated in characterization of field-grown plants and preparation of plant samples for cell wall analysis, analyzed the data, and wrote the manuscript. HB performed rust disease phenotyping and statistical analysis, and participated in preparation of plant samples for cell wall analysis. AS performed transgenic lines generation and phylogenetic tree work. GL and HX performed gene expression analysis. AD, MR, JN, and BD performed ethanol yield analysis. J-YZ and MU performed cloning of the target gene. GT, RS, and MD performed lignin and sugar release analyses. Z-YW produced the transgenic plants. YT performed RNA-seq analysis. EB and YT conceived of the experimental approach and made significant intellectual contributions about the target gene and cell wall biology. CS conceived of the field study and its design and coordination, and assisted with interpretation of results and revisions to the manuscript. All authors contributed to text and data analysis. All authors read and approved the final manuscript.

## Conflict of Interest

The authors declare that the research was conducted in the absence of any commercial or financial relationships that could be construed as a potential conflict of interest.

## Footnotes

1 https://phytozome-next.jgi.doe.gov/

2 https://www.genome.jp/kegg/kegg2.html

3 https://en.wikipedia.org/wiki/Anthocyanin#Biosynthesis
