# Supplementary material for: Silencing Folylpolyglutamate Synthetase1 (FPGS1) in Switchgrass (Panicum virgatum L.) Improves Lignocellulosic Biofuel Production
Source: Front Plant Sci. 2020 Jun 19;11:843. doi: 10.3389/fpls.2020.00843 (PMC7317012; doi:10.3389/fpls.2020.00843)
Supplement: Supplementary file 1 [file Data_Sheet_1.PDF]

GCCGGCGATGAGCTGGGTAAGAAAAGAAATAGGCCCACCAGTCAGCGAGCCGCCCTATGCCGCGCCCGTG
GGGCCCACCGCGGCGCCGCCGGCACGACCACCAACAGCGCCATCCCACCGCCGCCGCTGCAGCCCGCACC
CGCTCCACGCTTATGCGCTCGCGTCCCCCTCTCGCCGCCCACCTCCGCCGCCTCTTCCTCCTCCACCCAA
CTAACCCATCGCGCCGCC**ATGGCCTCCTCCACCGCCGCCGTCGCGCGGGCAGGTGCTGTGCCGGCGGCGG**
AGTACGAGGAGGCGCTGGGGCGCCTCTCCTCGCTCATCACGCAGAAGGTGCGCGCGCACAGCGGCAACCG
AGGCAACCAGTGGGACCTCATGGCACACTACGTCAAGATTCTGGAGCTGGAGGAGCCGATCGCGCGGATG
AAGGCCATCCACGTCGCAGGGACCAAGGGCAAGGGTTCGACATGCACATTTACTGAGTCAATCCTGCGAT
CGTGTGGCTTCCACACTGGGCTCTTCACCTCACCACATTTGATGGATGTCAGGGAGCGATTCCGGCTAGA
TGGAGTTGATATTTCTGAAGAGAAATTTTTGAACTACTTTTGGTGGTGCTGGAATAAGTTGAAGGAGAAG
ACTGACGATGATATTCCCATGCCAGCCTATTTCAGGTTCCTGGCGTTGCTCGCATTCAAGATATTTTCTG
CTGAGCAGGTAGATGTTGCTGTTCTCGAGGTTGGCCTTGGAGGGAAGTTTGATGCAACTAATGTGGTTGA
AGCACCTGTAGTTTGTGGGGTATCTTCGCTTGGATATGATCATATGGAAATTCTTGGGCATACGCTTGGA
GAAATCGCAGGGGAGAAGGCTGGGATCTTCAAGAAAGGAGTTCCAGCCTATACTGCTCCACAACAAGAAG
AGGCAATGGTTGCTCTCAAACAAAGAGCTTCGGAGTTGTGTATACCTCTCCAAGTTGCTGATCCTCTGGT
GCCGTATCACTTAAAAGGTCAACATCTTGGACTGAATGGTGAACACCAATACATAAATGCTGGCCTTGCT
GTTGCTTTGGCTAGTACATGGCTTGAGAAGCAGGGGCATAAGGACAGGATACCACTCAATCGTACTGACC
CCCTACCAGATCATTTTATTAGAGGTCTATCAAATGCTTGTTTGCAAGGGCGAGCACAGATTGTTCCAGA
TTCACAAGTGAATTCAGGAGCGGCCAGAAATTCTTCTTTGGTTTTCTATTTGGATGGGGCTCACAGTCCT
GAAAGTATGGAAATATGTGCCAAGTGGTTTTCCCATGTCACTAATGATGATAAAAGAATACCATCTTCCA
CAGAGCAGTCTCAGAGTTCGAAGTCTCTAAAGATCCTTCTGTTCAATTGCATGTCCGTGAGAGATCCTAT
GAGACTGCTTCCGCATCTCCTGGATGCCTCAACTCAAAATGGAGTCCACTTTGATCTGGCCCTATTTGTA
CCAAATCAATCGCAATACAACAAGCTTGGTTCTAGTACATCAGCACCTGCTGAGCCTGAACAAATCGATT
TGTCATGGCAGTTGTCACTCCAAACAGTGTGGGAGAAGTTACTTCAGGATAAAGGAATAGATAGTGCAAA
TTCCAGTGACAATAGTAAAGTTTTTGCATCTCTTCCACTTGCGATCGAGTGGCTAAGGAAGAATGCCCAA
GAAAACGGATCTACTTCTTTTCAGAACCAGGTCTTGGTTACTGGCTCCCTGCATCTTGTTGGTGATGTCT
TGAGGCTTATTAAGAAGTGATCTATTTGGAAGTGATATACAGTTTGAAGTTCATAGTGGAAGCAATATTT
GATGTGTCCCGGGGCTAACATGATTAGCAAGGGCAGAAATCTCAACGGCTTGATTATGATGCTAACCAAG
CTGCGAGGTTCTTGACTTGTACATTCTGTGTTTTCTGGCTACGGTTCGCAACCTACAGTCAGGGAATTTG
TGTGCCCACTGTTGTAGTTCAGAATGTAAACTCAGGAATAAGGTGATTTTGATCAGGCCATCATTGAACT
AGTTTGAGAAAATCAGAAAACTTGGAATGGAACTCGGTCTAGCAATAATTGAGCTTTGGTCTTTCTCAGT
ATTTGCGCTTGCAGCTAAACCAGTCCAATTATTACCATCAGATCTGTGATCGTTGCAGAATTCATTCATC
GGAAAGATAGCTGTAC

Figure S1. Sequence of the *PvFPGS1* cDNA fragment. The full-length coding sequence is bolded. The sequence selected for use in the RNAi cassette is underlined and highlighted.

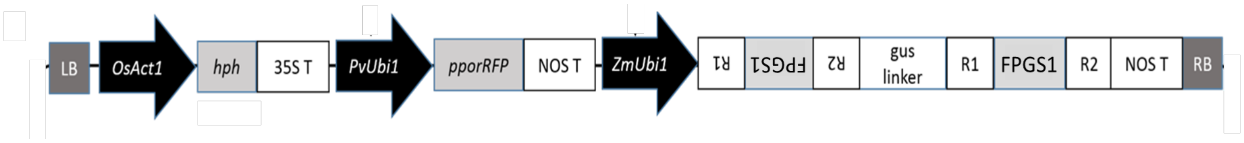

Figure S2. Schematic diagram of the pANIC8A plant RNAi-expression vector. Abbreviations: *OsAct1* (rice actin 1 promoter and intron), *hph* (hygromycin), 35S T (35S terminator), *PvUbi1* (switchgrass ubiquitin 1 promoter and intron), *pporRFP* (*Porites porites* red fluorescent protein), NOS T (nopaline synthase terminator), *ZmUbi1* (maize ubiquitin 1 promoter), *FPGS1* (*FPGS1* sequence fragment), R1 and R2 (*attR1* and *attR2* recombinase sites), gus linker (linker from *uidA* gene), LB (left border), RB (right border).

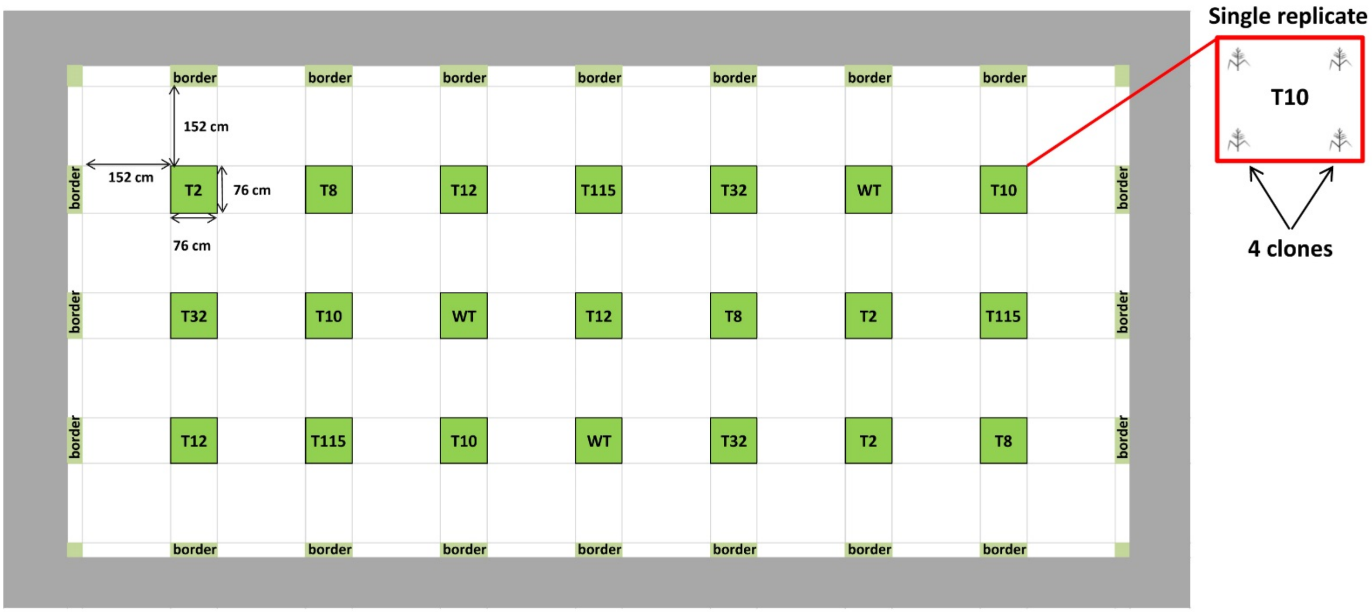

Figure S3. Field design of *FPGS1*-downregulated switchgrass. The field site was 24.2 m x 15.1 m. Transgenic (T2, T8, T10, T12, T32, and T115) and wild-type control (WT) replicates were arranged in a randomized complete block design (RCBD) and surrounded by a row of non-transgenic border plants. Three replicates were included for each transgenic and wild-type control lines. Each replicate contained four vegetatively-propagated clones of a single line. Replicates were 152 cm apart with 76 cm spacing among the four clones.

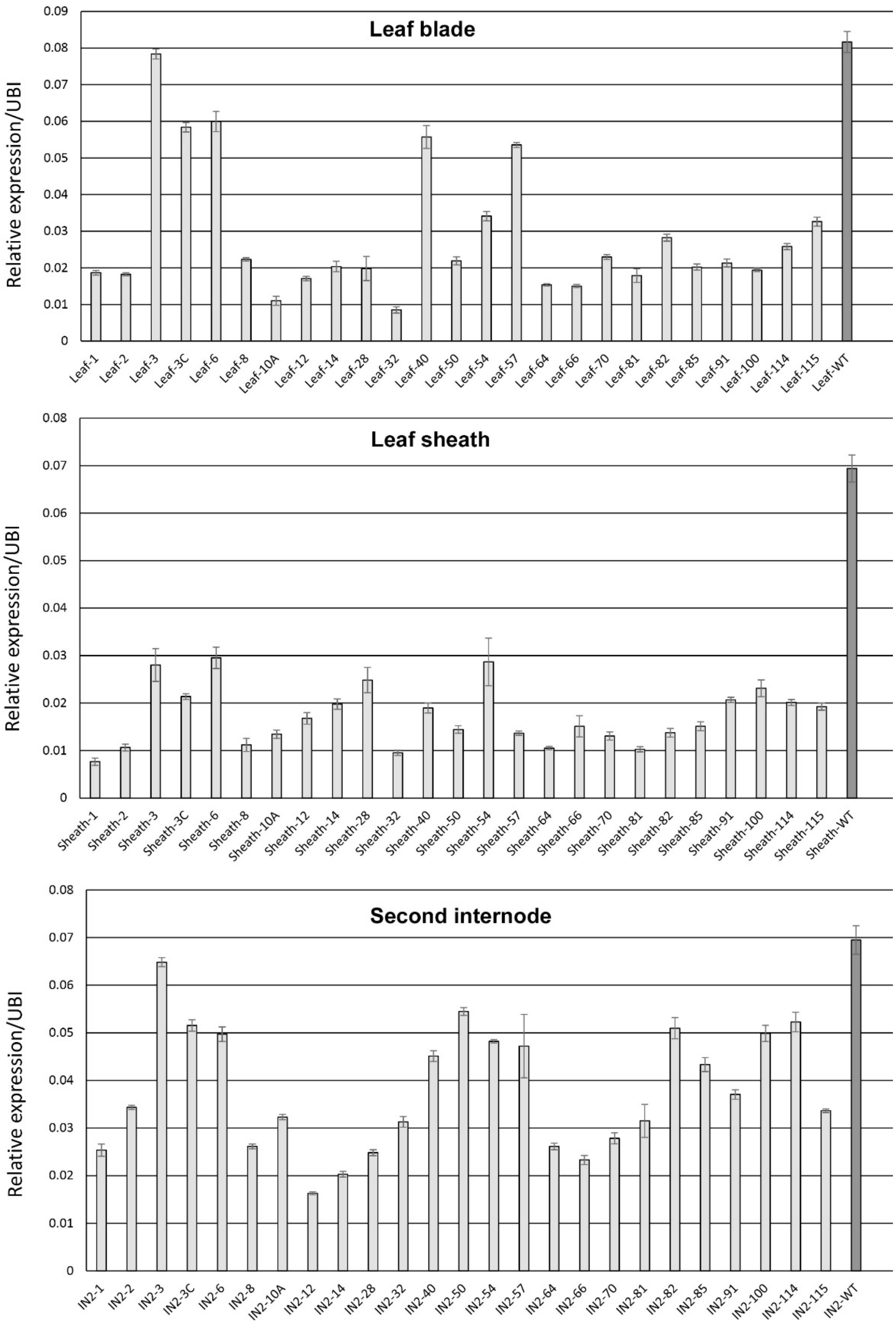

Figure S4. Relative transcript levels of *PvFPGS1* in RNAi-transgenic lines as determined by qRT-PCR. Plant samples for RNA extraction were collected from leaf blade, leaf sheath, and second internode at the R1 (reproductive stage 1) development stage. WT: non-transgenic control. The relative levels of transcripts were normalized to the switchgrass ubiquitin 1 gene expression (UBI). Bars represent mean values of three biological replicates ± standard error.

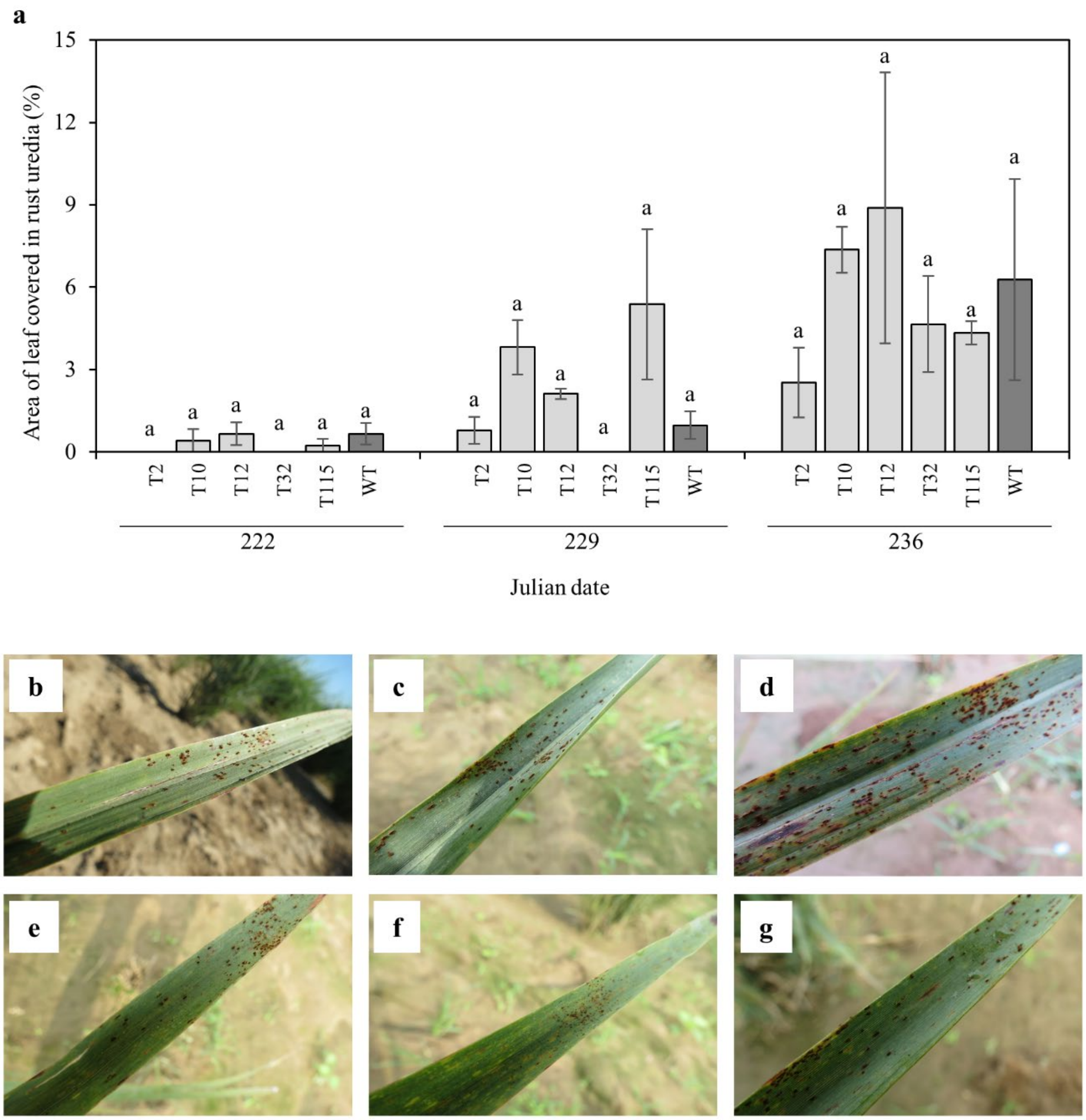

Figure S5. Rust (*P. novopanici*) disease severity in *FGPS1*-downregulated switchgrass in year two (2015) of the field experiment. (a) Rust severity as determined by the average percentage of the leaf covered in rust uredia. Bars are the mean of three biological replicates for each transgenic line (T2, T10, T12, T32, and T115) and wild type control (WT) ± standard error. Means were compared by a one-way ANOVA and letter groupings were obtained using Fisher's least significant difference method. There were no significant differences at the 5% level. (b-g) Images of rust symptoms on lines T2 (b), T10 (c), T12 (d), T32 (e), T115 (f), and the wild type control (g).

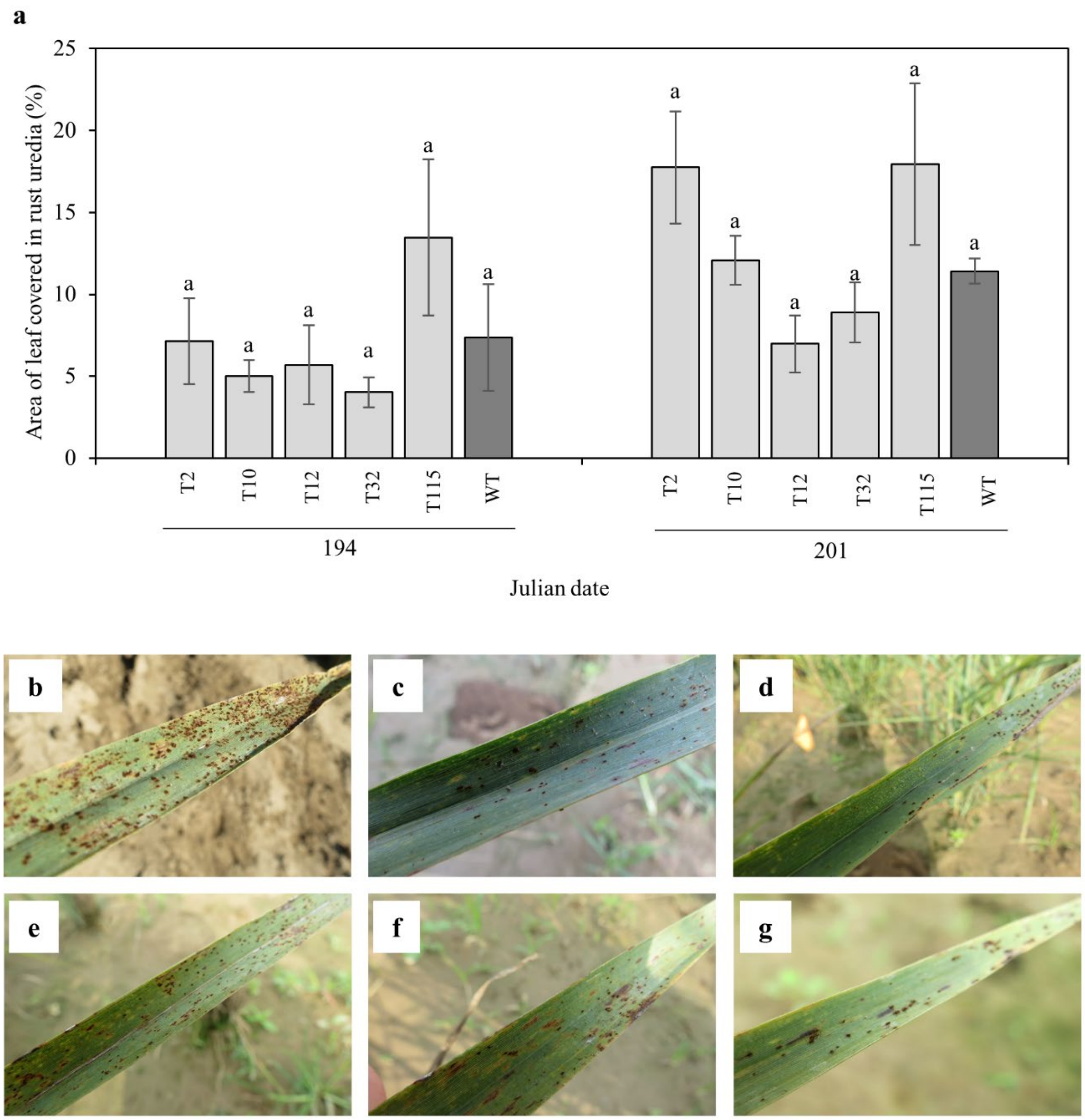

Figure S6. Rust (*P. novopanici*) disease severity in *FGPS1*-downregulated switchgrass in year three (2016) of the field experiment. (a) Rust severity as determined by the average percentage of the leaf covered in rust uredia. Bars are the mean of three biological replicates for each transgenic line (T2, T10, T12, T32, and T115) and wild type control (WT) ± standard error. Means were compared by a one-way ANOVA and letter groupings were obtained using Fisher's least significant difference method. There were no significant differences at the 5% level. (b-g) Pictures of rust symptoms in transgenic switchgrass lines T2 (b), T10 (c), T12 (d), T32 (e), T115 (f), and the wild type control (g).
